# DNA damage repair-related gene signatures delineate immune-distinct subtypes and diagnostic cues in tuberculosis

**DOI:** 10.3389/fcimb.2026.1885489

**Published:** 2026-09-25

**Authors:** Abdusemer Reyimu, Mamatali Rahman, Amina Abudurousuli, Xiang Cheng, Xiu Liu, Aimin Xu, Xiaoguang Zou

**Affiliations:** Department of Laboratory Medicine, The First People’s Hospital of Kashi, Xinjiang, Kashi, China

**Keywords:** compound screening, DNA damage repair, immune infiltration, machine learning, molecular subtypes, single-cell RNA sequencing, tuberculosis

## Abstract

**Background:**

DNA damage repair-related genes (DDR-RGs) may shape tuberculosis (TB) pathogenesis, but their molecular characteristics, diagnostic utility, and cellular context remain insufficiently defined.

**Methods:**

We integrated multiple GEO transcriptome datasets, identified differentially expressed genes, constructed weighted gene co-expression network analysis modules, and intersected these results with curated DDR-RGs to obtain candidate genes. An ensemble framework of 110 machine-learning model combinations was used to select diagnostic markers, followed by external validation, consensus clustering, immune infiltration analysis, single-cell RNA-seq evaluation, and Comparative Toxicogenomics Database-based compound-association analysis.

**Results:**

The integrated training cohort included 134 samples; 94 differentially expressed genes and a 392-gene key module were identified, yielding 25 candidate DDR-RGs. The Ridge + SVM model performed best and selected ETV7, STAT1, FBXO6, and TAP1 as core DDR-RGs, achieving AUCs of 0.991, 0.925, 0.939, and 0.981 in the training and three validation cohorts. Consensus clustering separated TB patients into two DDR-related subtypes, with Cluster 1 enriched in immune-related pathways and characterized by distinct immune-cell infiltration. Single-cell analysis revealed cell-type-specific expression of core DDR-RGs in pulmonary TB, and compound-association analysis identified 35 candidate compounds, including six associated with multiple core genes.

**Conclusion:**

These findings characterize DDR-related molecular heterogeneity in TB and support ETV7, STAT1, FBXO6, and TAP1 as candidate diagnostic biomarkers and exploratory candidates for host-directed studies.

## 1 Introduction

Tuberculosis (TB), a chronic infectious disease caused by Mycobacterium tuberculosis (Mtb), remains a major global public health challenge. According to the World Health Organization Global tuberculosis report 2025, an estimated 10.7 million people developed TB in 2024 and 1.23 million people died from the disease, keeping TB among the leading causes of death from infectious diseases worldwide (World Health Organization, 2025). Although the Bacillus Calmette-Guérin (BCG) vaccine reduces the risk of severe TB in children, its protective efficacy against adult TB remains limited (Mangtani et al., 2014). As an intracellular pathogen, Mtb can promote genomic instability in host macrophages (Castro-Garza et al., 2018) and may induce multiple forms of host DNA damage, including oxidative DNA damage and double-strand breaks. Host DNA damage response and repair mechanisms are essential for maintaining genomic stability and directly influence cell fate, including survival, senescence, and apoptosis, as well as the efficiency of immune responses (Sancar et al., 2004; Jackson and Bartek, 2009). In parallel, the bacterial DNA repair system helps Mtb withstand DNA damage imposed by host immune responses, such as reactive oxygen species and reactive nitrogen species (Dos Vultos et al., 2009; Reiche et al., 2017). Therefore, systematically investigating TB from the perspective of DNA damage repair (DDR) may help identify diagnostic markers and host-response pathways relevant to future translational studies.

The development and progression of TB are closely associated with disruption of host genomic stability (Castro-Garza et al., 2018). DDR comprises a series of molecular processes through which cells detect and correct DNA lesions, including base excision repair (BER), nucleotide excision repair (NER), mismatch repair (MMR), homologous recombination repair (HR), and non-homologous end joining (NHEJ) (Sancar et al., 2004; Jackson and Bartek, 2009). These pathways coordinately repair diverse forms of DNA damage, including oxidative damage, single-strand breaks, and double-strand breaks (Sancar et al., 2004; Jackson and Bartek, 2009). DDR plays a dual role in anti-TB immunity: appropriate repair maintains immune-cell function and prevents excessive cell death, whereas insufficient or dysregulated repair may lead to genomic instability, cellular senescence, or aberrant proliferation, thereby reshaping the immune microenvironment (Sancar et al., 2004; Jackson and Bartek, 2009). In addition, Mtb relies on its own repair machinery to resist host immune pressure and maintain intracellular survival (Dos Vultos et al., 2009; Brzostek et al., 2014; Szulc-Kielbik et al., 2015; Reiche et al., 2017).

Previous studies have shown that Mtb infection can induce genomic instability in host macrophages and can create DNA-damaging stress in mycobacteria (Dos Vultos et al., 2009; Reiche et al., 2017; Castro-Garza et al., 2018). Certain Mtb-associated stress-response pathways may intersect with host apoptosis-related signaling; however, the precise relationship between these bacterial factors and p53-mediated apoptosis in TB remains incompletely defined. Moreover, Mtb DNA repair enzymes and repair pathways, including RecA-dependent responses and DSB repair through NHEJ or HR, are important for bacterial stress resistance and survival in macrophage-related infection models (Brzostek et al., 2014; Szulc-Kielbik et al., 2015). DNA damage and oxidative stress in TB-related contexts can be reflected by markers such as phosphorylated histone H2AX (γ-H2AX) and 8-hydroxy-2’-deoxyguanosine (8-OHdG), whereas canonical DDR pathways provide the molecular basis for repairing such lesions (Sancar et al., 2004; Jackson and Bartek, 2009; Castro-Garza et al., 2018). These findings suggest that molecules involved in DDR may serve as potential diagnostic or disease-monitoring markers.

Despite these advances, comprehensive and integrative investigations of DNA damage repair-related genes (DDR-RGs) in TB remain limited. Most previous studies have focused on individual repair pathways or specific repair enzymes, often overlooking the broader regulatory framework of DDR-RG networks. Furthermore, systematic analyses of DDR-RG expression profiles based on large-scale transcriptome data, as well as the identification of DDR-related molecular subtypes, are still lacking.

DDR comprises multiple interconnected pathways, including BER, NER, MMR, HR, and NHEJ, together with numerous repair proteins and cell-cycle checkpoint regulators, forming a highly coordinated regulatory network (Sancar et al., 2004; Jackson and Bartek, 2009). Traditional single-gene approaches are insufficient to clarify the global role of this network in TB. Therefore, in the present study, we employed a multi-dataset integration strategy combined with machine learning algorithms to systematically investigate the transcriptional characteristics of DDR-RGs in the blood of TB patients. We further used single-cell RNA sequencing (scRNA-seq) data to characterize cell-type-specific expression patterns of core DDR-RGs at single-cell resolution and performed exploratory compound screening to assess reported gene-compound associations. Specifically, this study aimed to: (1) identify key diagnostic markers associated with DDR; (2) perform molecular subtyping of TB patients based on core DDR-RGs; (3) analyze the immune infiltration characteristics and biological features of different subtypes; and (4) evaluate the diagnostic value and reported compound associations of core DDR-RGs through multidimensional analyses. Through these efforts, we sought to provide evidence supporting future studies on TB diagnosis, molecular stratification, and host-response-guided research. The overall analytical workflow of this study is shown in **Figure 1**.

**Figure 1:**
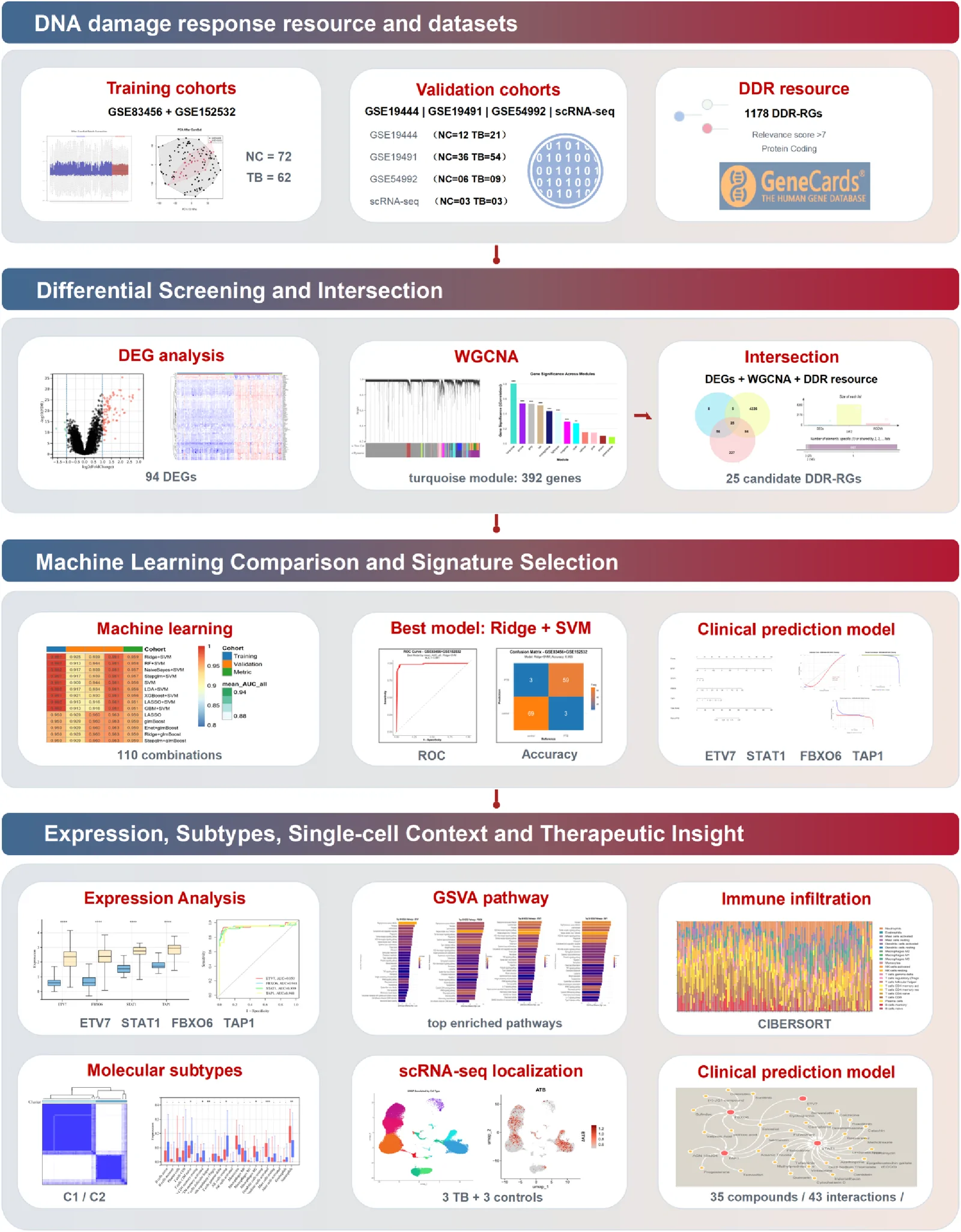
Analytical workflow of the study.

## 2 Materials and methods

### 2.1 Data acquisition

In R software (version 4.4.1), five public human PBMC transcriptome datasets related to TB were retrieved from the Gene Expression Omnibus (GEO) database: GSE83456, GSE152532, GSE19444, GSE19491, and GSE54992 (**Supplementary Table S1**) (Barrett et al., 2013; R Core Team, 2024). Datasets were included when active TB and healthy control samples, clear phenotype annotations, series matrix files or raw expression data, and corresponding platform annotation files were available. Datasets lacking healthy controls, complete phenotype information, or blood-derived samples were excluded. Five GEO datasets met these criteria. GSE83456 and GSE152532 were generated on GPL10558 and were integrated using ComBat from the sva package (version 3.52.0) to construct the GSE83456_GSE152532_ComBat training cohort (Johnson et al., 2007; Leek et al., 2012). GSE19444 and GSE19491 were generated on GPL6947, and GSE54992 was generated on GPL570; these datasets were used as independent validation cohorts. Dataset platforms, sample sizes, sample sources, and analytical roles are summarized in **Supplementary Table S1**. Series matrix files were used to construct probe-level expression matrices. The need for normalization was assessed according to sample medians, distribution skewness, and global range. For datasets showing distributional differences, quantile normalization was performed with the limma package (version 3.60.4); box plots and density plots were used for quality control (Ritchie et al., 2015). Probe identifiers were converted to gene symbols using platform annotation files, and the average value was calculated for genes corresponding to multiple probes. Missing values were handled in two steps: genes with missing rates > 50% were first removed, and the remaining missing values were then imputed using k-nearest neighbor (KNN) imputation (impute.knn, k = 10) (Troyanskaya et al., 2001). DDR-RGs were retrieved from the GeneCards database (https://www.genecards.org/) using a relevance score > 7, yielding 1,178 DDR-RGs for subsequent analysis (**Supplementary Table S2**) (Stelzer et al., 2016).

### 2.2 Differential gene expression selection

Differential expression analysis was performed on the merged training cohort (GSE83456_GSE152532_ComBat) using the limma package (version 3.60.4) in R (Ritchie et al., 2015). Samples were classified into a healthy control group (72 cases) and a TB group (62 cases), and a corresponding design matrix was constructed. Linear models were fitted using lmFit, followed by empirical Bayes moderation with eBayes (Ritchie et al., 2015). P values were adjusted using the Benjamini-Hochberg procedure, and the adjusted P value was reported as adj.P.Val in the limma output (Benjamini and Hochberg, 1995). Genes with |log_2_ fold change| > 1 and adj.P.Val < 0.05 were defined as differentially expressed genes (DEGs). Differential expression results were visualized using heatmaps generated with the gplots package (version 3.1.3.1) and volcano plots generated using base plotting functions.

### 2.3 Weighted gene co-expression network analysis (WGCNA)

WGCNA was applied to the training cohort for co-expression module detection and module-trait correlation analysis (Langfelder and Horvath, 2008). The median absolute deviation (MAD) of each gene was calculated, and the 50% of genes with the lowest MAD values were removed. The goodSamplesGenes function was used to exclude outlier samples and genes. Candidate soft-thresholding powers were evaluated based on the scale-free topology criterion. Network construction was performed with networkType = “unsigned”. According to the WGCNA soft-thresholding procedure, β = 14 was used for the unsigned network. At this power, the scale-free topology fit index was 0.87. A Pearson correlation matrix was then converted into a weighted adjacency matrix using β = 14. The adjacency matrix was transformed into a topological overlap matrix (TOM), and 1 − TOM was used as the dissimilarity measure for average linkage hierarchical clustering. Modules were identified using the dynamic tree-cutting method with the software argument deepSplit = 3 and a minimum module size of 50 genes. Similar modules were merged when eigengene dissimilarity was < 0.25. Candidate DDR-RGs were defined as genes overlapping among DEGs, the key WGCNA module, and the curated DDR-RG set.

### 2.4 Selection of core DDR-RGs

A multi-algorithm modeling framework was used to identify diagnostic markers for TB and establish a predictive model. After merging datasets and extracting shared genes, expression values were standardized using the scale function for Z-score normalization. Candidate genes were ranked according to random forest importance (MeanDecreaseAccuracy) using the randomForest package (version 4.7.1.2) (Breiman, 2001; Hastie et al., 2009). Gene combinations were then generated incrementally according to importance ranking, and the optimal gene set was selected by maximizing the average area under the receiver operating characteristic curve (AUC) across validation cohorts. The modeling framework incorporated 11 machine-learning algorithms: elastic net, Ridge, stepGLM, LASSO, SVM, LDA, RF, GBM, XGBoost, Naive Bayes, and glmBoost (Cortes and Vapnik, 1995; Tibshirani, 1996; Breiman, 2001; Friedman, 2001; Zou and Hastie, 2005; Bühlmann and Hothorn, 2007; Hastie et al., 2009; Chen and Guestrin, 2016). Model construction used glmnet 4.1.8, e1071 1.7.16, MASS 7.3.60.2, gbm 2.2.2, xgboost 1.7.11.1, mboost 2.9.11, and caret 7.0.1. These algorithms were cross-combined as feature selectors and classifiers to generate 110 model combinations. All models were trained and tuned using 3-fold cross-validation, and their generalization performance was evaluated across independent datasets. AUC was used as the primary evaluation metric, and the performance of each model in the training and validation cohorts was summarized using heatmaps. The optimal model was selected according to the average AUC across validation cohorts. Receiver operating characteristic (ROC) curves and confusion matrices were generated for the best-performing model, and sensitivity, specificity, and balanced accuracy were calculated to assess diagnostic performance.

### 2.5 Expression and diagnostic value of core DDR-RGs

Volcano plots for core DDR-RGs were generated with ggplot2 (version 3.5.1) (Wickham, 2016). GeneMANIA was used for gene-interaction and functional annotation analysis with an interaction score threshold > 0.4 (Warde-Farley et al., 2010). Expression differences between TB patients and healthy controls were compared using box plots. ROC curves were generated using the pROC package (version 1.19.0.1), and AUC values were calculated to assess diagnostic performance (Robin et al., 2011). The same analyses were repeated in independent validation cohorts.

### 2.6 TB diagnostic model construction and validation

A logistic regression model was developed using the rms package (version 8.1.0) in the GSE83456_GSE152532_ComBat training cohort, and a nomogram was constructed (Harrell, 2015). In the nomogram, each gene was assigned a score, and the total score was used to estimate TB risk. Model calibration was assessed using calibration curves, including the intercept, slope, and Brier score (Harrell, 2015). Clinical utility was evaluated using decision curve analysis (DCA) and clinical impact curves (Vickers and Elkin, 2006). AUC values with 95% CI were calculated in the training and validation cohorts.

### 2.7 Gene set variation analysis (GSVA)

KEGG pathway activity was quantified with the GSVA package (version 1.52.3) (Hänzelmann et al., 2013; Kanehisa et al., 2023). Pathway gene sets were obtained from the Kyoto Encyclopedia of Genes and Genomes (KEGG) database and filtered by size before analysis (Kanehisa et al., 2023). GSVA scores were calculated for each sample in the merged dataset (Hänzelmann et al., 2013). Samples were categorized into high- and low-expression groups according to the median expression of each core gene. A t-test with FDR correction was used to identify pathways with significant differences in activity between groups (Benjamini and Hochberg, 1995). Significant pathways were illustrated using bar plots, and heatmaps of immune-related pathways were generated to characterize the potential biological functions of the core DDR-RGs in TB.

### 2.8 Immune infiltration analysis

The CIBERSORT algorithm was used to quantitatively assess differences in immune cell infiltration associated with TB (Newman et al., 2015). Based on the LM22 leukocyte gene signature matrix, CIBERSORT estimates the proportions of 22 immune cell types from bulk gene expression data by deconvolution (Newman et al., 2015). Immune-cell composition was inferred from the bulk transcriptomic profiles of the GEO cohorts. The analysis used the preprocessed GSE83456_GSE152532_ComBat dataset, comprising 134 samples, including 62 TB cases and 72 healthy controls. Genes with an average log_2_ expression value < 0.5 were excluded before analysis. CIBERSORT was run with 100 permutations (Newman et al., 2015). Differences in immune cell proportions between groups were assessed using the Wilcoxon rank-sum test, with P < 0.05 considered statistically significant. Bar plots and violin plots generated using ggplot2 (version 3.5.1) were used to visualize immune infiltration patterns (Wickham, 2016).

### 2.9 Molecular subtype identification of TB based on core DDR-RGs

Consensus clustering analysis was performed to classify TB patients based on the expression profiles of the four core DDR-RGs (ETV7, STAT1, FBXO6, and TAP1). Expression data from 62 TB samples in the GSE83456_GSE152532_ComBat training cohort were analyzed using the ConsensusClusterPlus package (version 1.58.0) (Wilkerson and Hayes, 2010). Cluster stability was evaluated using the resampling framework in ConsensusClusterPlus. Parameters were set as follows: maxK = 6, reps = 50, pItem = 0.8, pFeature = 1, clustering algorithm = partitioning around medoids (PAM), and distance metric = Euclidean distance. The optimal number of clusters was determined according to the cumulative distribution function (CDF) curve, delta area plot, and consensus score, and k = 2 was selected. Uniform manifold approximation and projection (UMAP) was used to visualize subtype separation (McInnes et al., 2018). Differences in core DDR-RG expression between subtypes were evaluated using the Wilcoxon rank-sum test, and heatmaps were generated using the pheatmap package (version 1.0.12).

### 2.10 Single-cell RNA sequencing

Peripheral blood samples from three patients with pulmonary TB and three healthy controls were analyzed by single-cell RNA sequencing. Raw sequencing data were processed with Cell Ranger to generate gene-cell expression matrices (10x Genomics, 2026). Downstream single-cell analyses were performed using Seurat (version 5.1.0), including quality control, normalization, highly variable gene selection, data scaling, batch correction, PCA, clustering, and UMAP visualization (Blondel et al., 2008; McInnes et al., 2018; Stuart et al., 2019; Hao et al., 2021). Cells were clustered using the Louvain graph-based algorithm. Cell types were annotated using SingleR (version 2.6.0) and manually curated according to canonical marker genes visualized by FeaturePlot and DotPlot (Aran et al., 2019). The annotated single-cell dataset was used for downstream cell-type-specific expression analysis of core DDR-RGs.

### 2.11 Compound-association analysis

To explore reported gene-compound associations involving core DDR-RGs, we searched the Comparative Toxicogenomics Database (CTD; http://ctdbase.org/; accessed March 15, 2026) (Davis et al., 2025). Reported chemical-gene interactions for the core DDR-RGs were retrieved from CTD. During screening, decreases/increases and activity/binding/expression/abundance were selected under Interaction Types, and protein/gene/mRNA were selected under Gene Form. Candidate results were subsequently evaluated according to compound type, interaction directness, and pharmacological relevance. Cytoscape was used to visualize gene-compound interaction networks (Shannon et al., 2003).

### 2.12 Statistical analysis

Continuous variables between two groups were compared using the Wilcoxon rank-sum test. All statistical analyses were performed using R software (version 4.4.1), and a two-sided P < 0.05 was considered statistically significant (R Core Team, 2024).

### 2.13 Software, databases, and reproducibility

The analysis was performed with R software version 4.4.1. Recorded package versions were limma 3.60.4, sva 3.52.0, impute 1.78.0, gplots 3.1.3.1, ggplot2 3.5.1, pROC 1.19.0.1, rms 8.1.0, GSVA 1.52.3, pheatmap 1.0.12, Seurat 5.1.0, SingleR 2.6.0, randomForest 4.7.1.2, e1071 1.7.16, glmnet 4.1.8, MASS 7.3.60.2, gbm 2.2.2, xgboost 1.7.11.1, mboost 2.9.11, and caret 7.0.1. These packages were used for batch correction, differential expression analysis, co-expression network construction, diagnostic modeling, pathway scoring, immune deconvolution, single-cell analysis, gene-network annotation, and gene-compound association screening.

## 3 Results

### 3.1 Identification of differentially expressed genes

Batch correction using ComBat was performed for GSE83456 and GSE152532. Box plots showed that the expression distributions became more comparable after batch correction (**Figures 2A, B**), and PCA analysis further supported the reduction of batch-related separation between datasets (**Supplementary Figure S1**). After integration, the training cohort comprised 62 TB samples and 72 healthy control samples. Using the criteria of |log_2_ fold change| > 1 and adj.P.Val < 0.05 after Benjamini-Hochberg correction, a total of 94 DEGs were identified, including 89 upregulated genes and 5 downregulated genes (**Supplementary Table S3**; **Figure 2C**). A heatmap of the top 50 DEGs is shown in **Figure 2D**.

**Figure 2:**
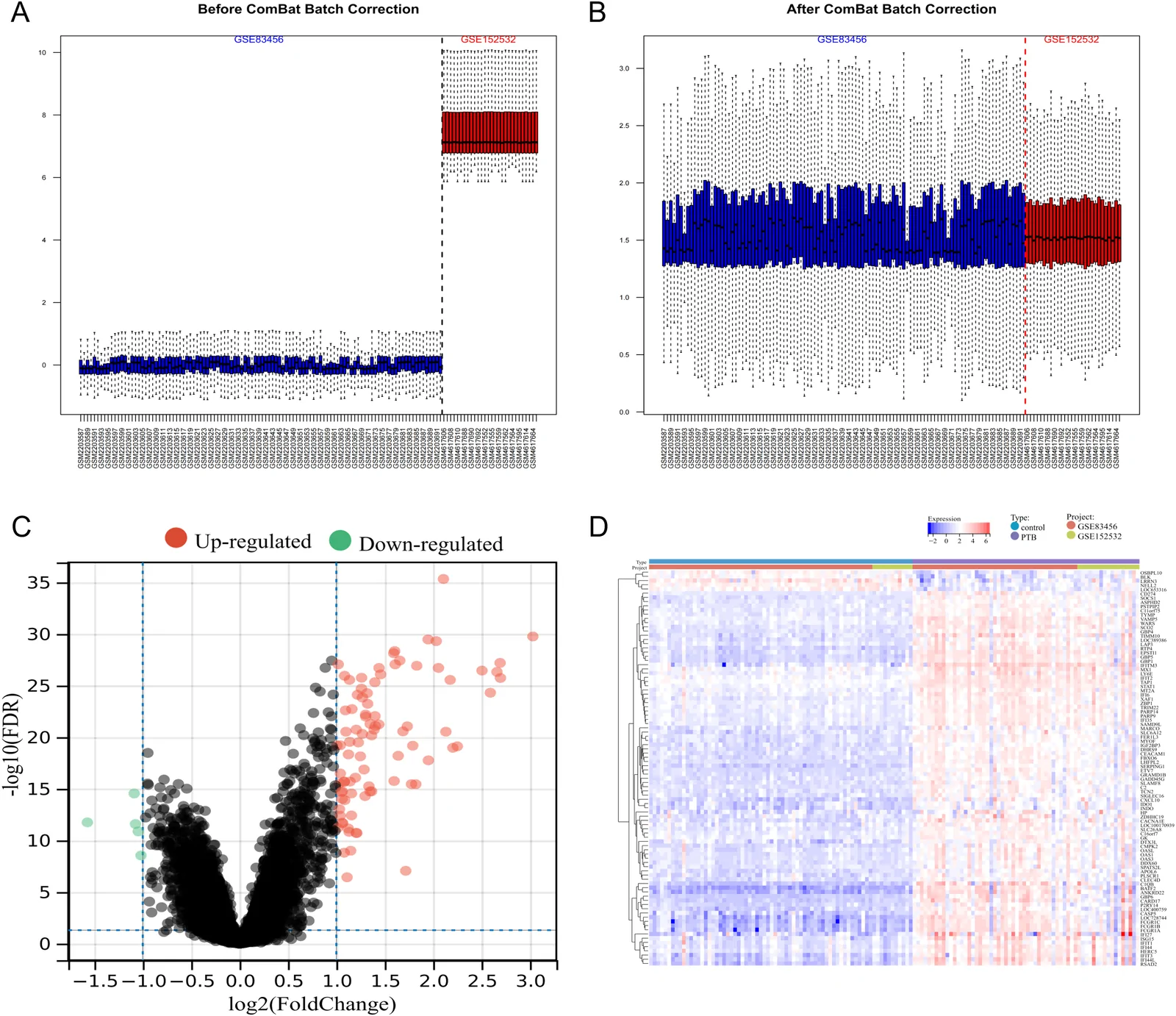
Analysis of differentially expressed genes (DEGs) between tuberculosis (TB) and healthy control samples. **(A, B)** Box plots showing the distribution of gene expression values in the GSE83456 and GSE152532 datasets before **(A)** and after **(B)** batch correction using ComBat. **(C)** Volcano plot of DEGs, with red and green dots indicating significantly upregulated and downregulated genes, respectively, and black dots indicating non-significant genes. **(D)** Heatmap of the top 50 DEGs showing distinct expression patterns between TB and control samples.

### 3.2 Construction of the TB co-expression network and identification of candidate DDR-RGs

To characterize phenotype-associated co-expression structure in the merged training cohort, WGCNA was performed. Candidate soft-thresholding powers and the corresponding mean connectivity profile are shown in **Figures 3A, B**. A soft-thresholding power of β = 14 was used for the unsigned network, with a scale-free topology fit index of 0.87 at this power (**Figure 3A**). The gene dendrogram and module assignment are shown in **Supplementary Figure S2**. A total of 12 co-expression modules were identified; the turquoise module showed the strongest correlation with the TB phenotype (r = 0.80, P = 1.5e-31) and contained 392 genes (**Supplementary Table S4**; **Figures 3C, D**). Genes within this module exhibited strong co-expression, with a correlation coefficient of 0.89 (P = 3.9e-138) between module membership and gene significance (**Figure 3E**). Intersecting DEGs, the curated DDR-RG set, and genes in the turquoise module yielded 25 candidate DDR-RGs (**Figure 3F**).

**Figure 3:**
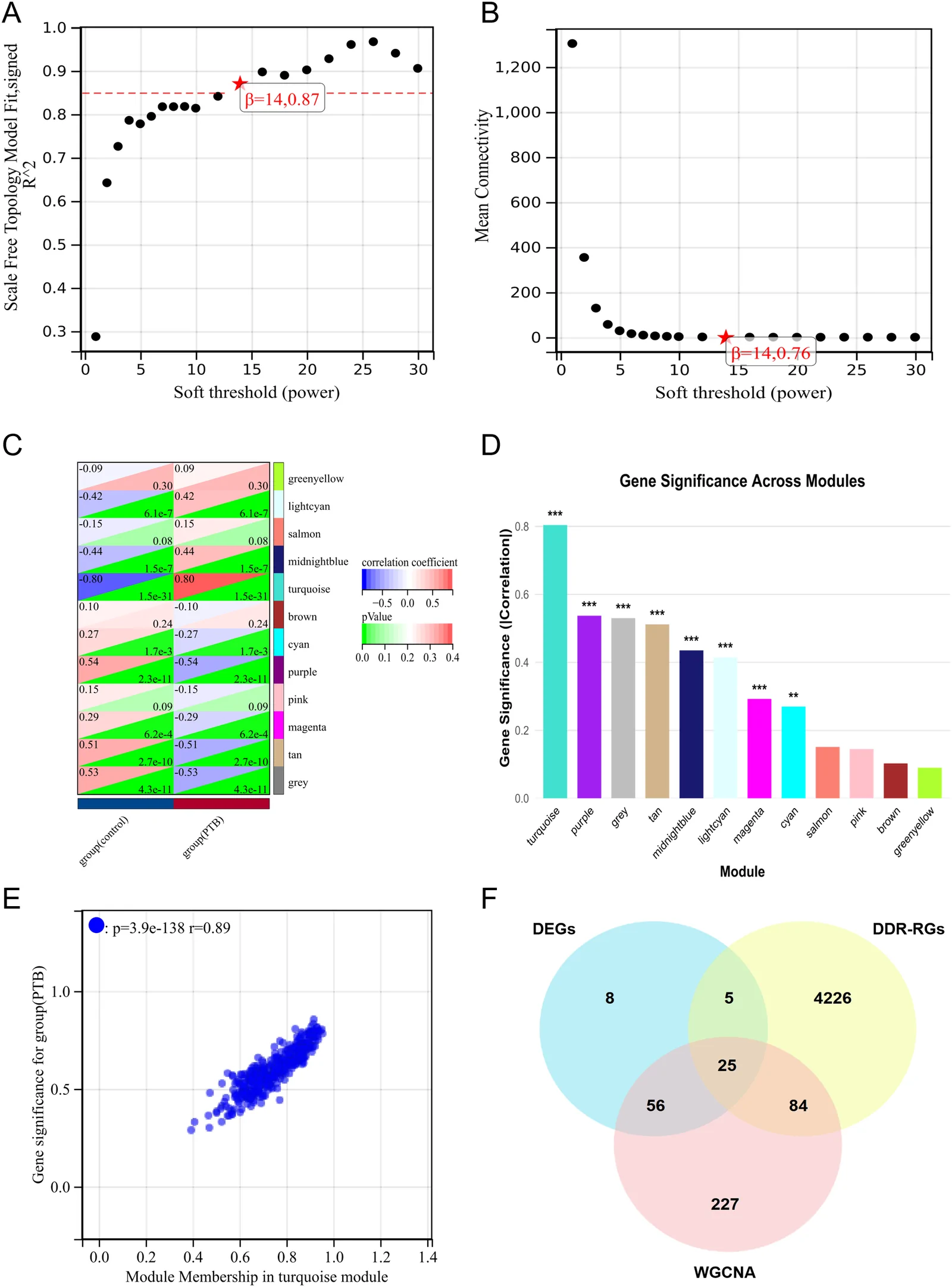
Weighted gene co-expression network analysis (WGCNA) and identification of candidate DNA damage repair-related genes (DDR-RGs). **(A)** Soft-threshold analysis for selection of the power used to construct the weighted co-expression network. The red dashed horizontal line indicates a reference scale-free topology fit level; β = 14 was used for the unsigned network and achieved a scale-free topology fit index of 0.87. **(B)** Mean connectivity under different soft-thresholding powers. **(C)** Correlation heatmap of module eigengenes and tuberculosis (TB) phenotype. **(D)** Bar plot of module-trait correlations highlighting the strong association between the turquoise module and TB. **(E)** Scatter plot of module membership versus gene significance in the turquoise module (correlation coefficient = 0.89, P = 3.9e-138). **(F)** Venn diagram showing the overlap among differentially expressed genes (DEGs), DDR-RGs, and genes in the key WGCNA module.

### 3.3 Selection of core DDR-RGs

By systematically comparing 110 model combinations generated from 11 machine learning algorithms using 3-fold cross-validation, we identified the optimal diagnostic model for DDR-RGs. The results showed that Ridge + SVM had the highest predictive performance in both the training and validation cohorts (**Figure 4A**). Based on this model, four core DDR-RGs were identified: ETV7, STAT1, FBXO6, and TAP1. ROC analysis showed that the optimal model achieved AUC values of 0.991, 0.925, 0.939, and 0.981 in the training cohort and three validation cohorts, respectively (**Figures 4B–E**). Model accuracy ranged from 80% to 100% across the four datasets (**Figures 4F–I**), indicating strong diagnostic performance and generalizability.

**Figure 4:**
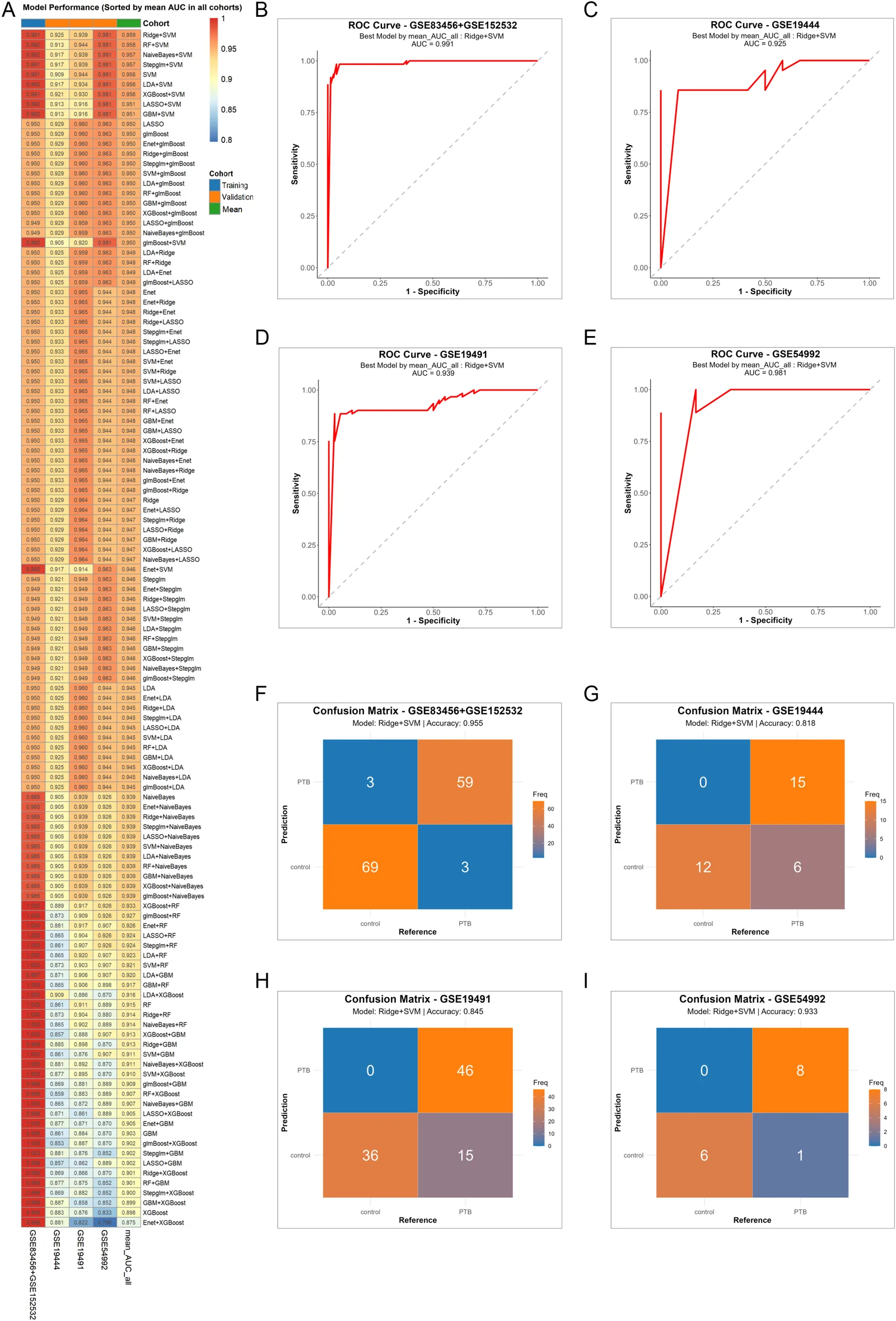
Identification of core DNA damage repair-related genes (DDR-RGs) by machine learning. **(A)** Performance of 110 model combinations evaluated by 3-fold cross-validation. **(B–E)** Receiver operating characteristic (ROC) curves and corresponding area under the receiver operating characteristic curve (AUC) values of the optimal model in the training cohort **(B)** and three validation cohorts **(C–E)**. **(F–I)** Confusion matrices of the optimal model in the training cohort and validation cohorts showing classification accuracy and misclassification rates.

### 3.4 Expression and diagnostic value of core DDR-RGs

Volcano plot analysis showed clear upregulation of the four core DDR-RGs in TB samples (**Figure 5A**). GeneMANIA analysis suggested that these genes were mainly associated with the cellular response to interferon-gamma, response to interferon-gamma, integral component of the endoplasmic reticulum membrane, antigen processing and presentation of exogenous peptide antigen via major histocompatibility complex (MHC) class I, phagocytic vesicles, regulation of innate immune responses, and MHC protein binding (**Figure 5B**). Box plot analysis showed that the expression levels of these four genes were significantly higher in TB patients than in healthy controls (**Figure 5C**). These findings were further validated in the independent cohorts GSE19444, GSE19491, and GSE54992 (**Figures 5E, G, I**). In the training cohort, the AUC values for ETV7, STAT1, FBXO6, and TAP1 were 0.950, 0.948, 0.939, and 0.940, respectively. In all validation cohorts, the AUC values for each gene exceeded 0.800, supporting their robustness as diagnostic biomarkers for TB (**Figures 5D, F, H, J**).

**Figure 5:**
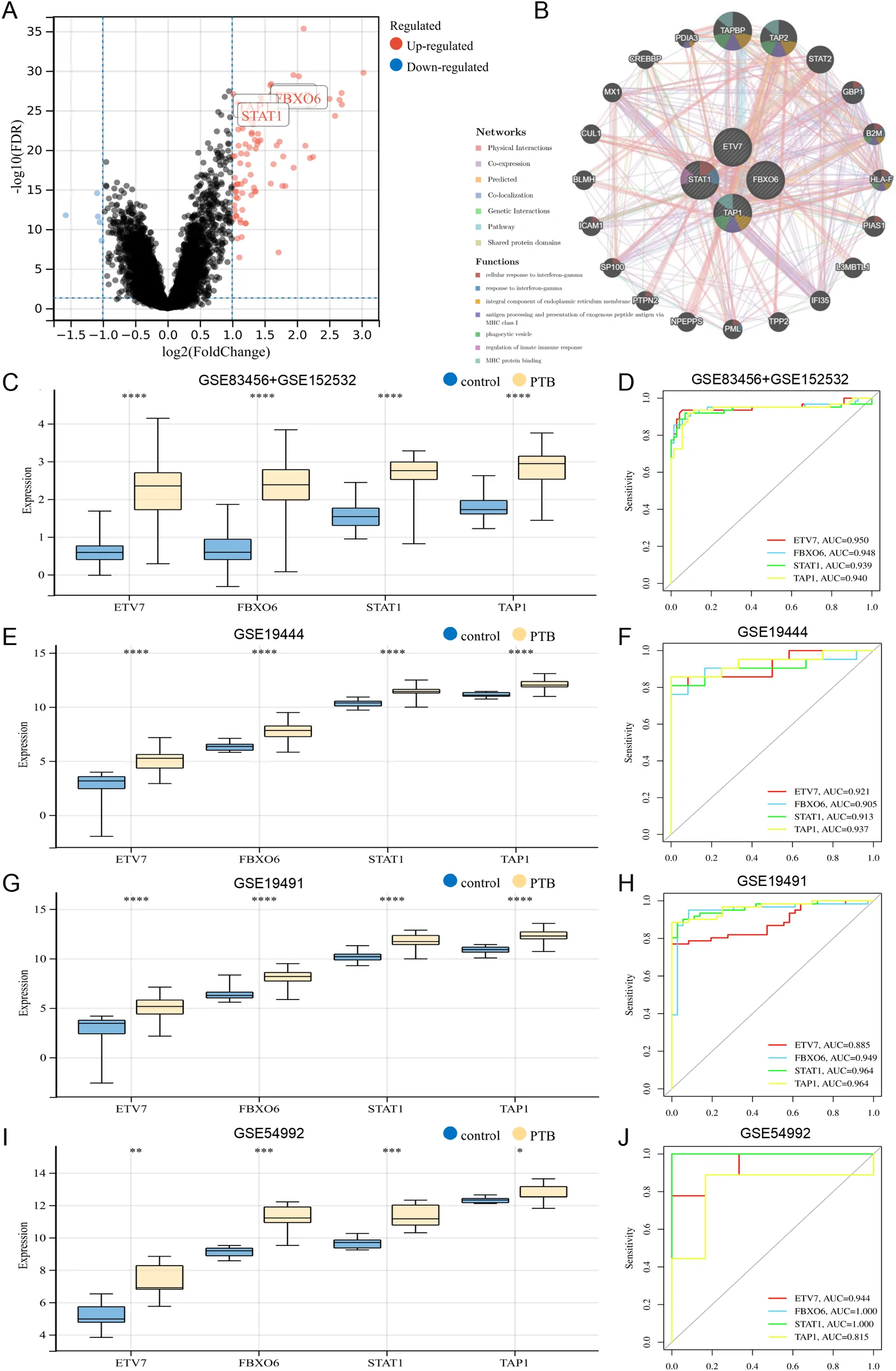
Expression and diagnostic evaluation of the core DNA damage repair-related genes (DDR-RGs). **(A)** Volcano plot showing the expression patterns of the four core DDR-RGs in tuberculosis (TB) and control samples. **(B)** GeneMANIA interaction network and functional annotation of the core DDR-RGs. **(C, E, G, I)** Box plots showing expression differences of the core DDR-RGs in the training cohort **(C)** and validation cohorts **(E, G, I)**. **(D, F, H, J)** Receiver operating characteristic (ROC) curves and area under the receiver operating characteristic curve (AUC) values of the core DDR-RGs in the training cohort **(D)** and validation cohorts **(F, H, J)**. *P < 0.05, **P < 0.01, ***P < 0.001, and ****P < 0.0001.

### 3.5 Clinical prediction model construction

A nomogram based on the four core DDR-RGs was constructed to facilitate individualized prediction of TB risk (**Figure 6A**). Calibration analysis showed good agreement between predicted probabilities and observed outcomes (**Figure 6B**). Decision curve analysis showed favorable net clinical benefit across a range of threshold probabilities (**Figure 6C**). The clinical impact curve further supported the predictive utility of the nomogram (**Figure 6D**).

**Figure 6:**
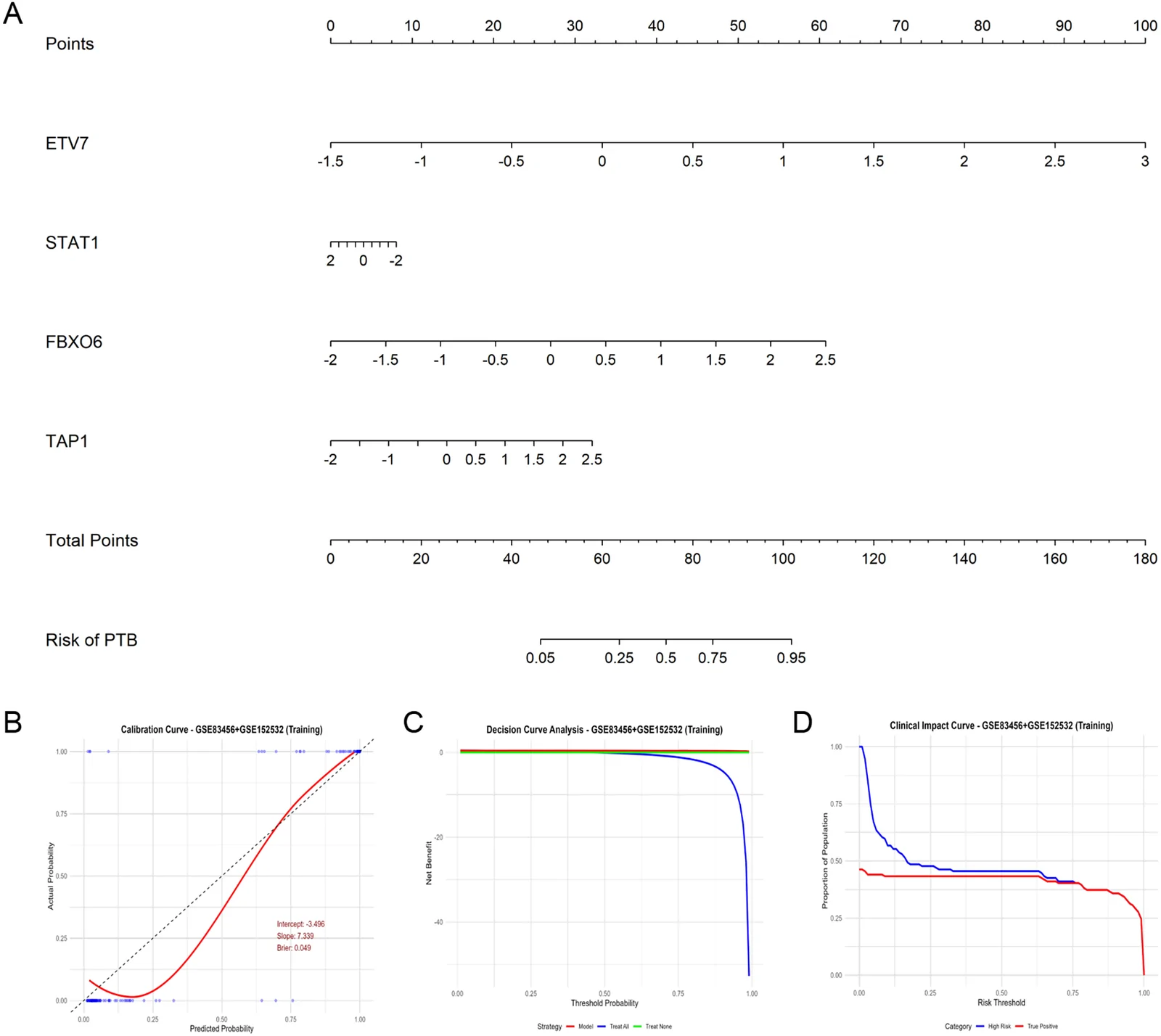
Development and validation of a clinical prediction model based on the core DNA damage repair-related genes (DDR-RGs). **(A)** Nomogram integrating ETV7, STAT1, FBXO6, and TAP1 for quantitative prediction of tuberculosis (TB) risk. **(B)** Calibration curve showing agreement between predicted and observed probabilities. **(C)** Decision curve analysis (DCA) showing the net clinical benefit of the model at different threshold probabilities. **(D)** Clinical impact curve showing the number of individuals classified as high risk and the number of true-positive events across threshold probabilities.

### 3.6 Enrichment analysis of core DDR-RGs

cGSVA based on KEGG pathways was summarized at the pathway level to emphasize collective downstream pathway alterations associated with the four core DDR-RGs (**Figures 7A–C**). Recurrent immune- and infection-related pathways included phagosome, PPAR signaling pathway, NOD-like receptor signaling pathway, Toll-like receptor signaling pathway, tuberculosis, necroptosis, antigen processing and presentation, Influenza A, Staphylococcus aureus infection, pertussis, herpes simplex virus 1 infection, and leishmaniasis (**Figures 7A, B**). Among significant KEGG pathways, 20 were associated with all four core DDR-RGs, whereas 8, 4, and 8 pathways were associated with one, two, and three core genes, respectively (**Figure 7C**). Together, the pathway-level GSVA summary showed that the core DDR-RGs were linked to shared immune and inflammatory pathway programs rather than isolated gene-specific pathway changes.

**Figure 7:**
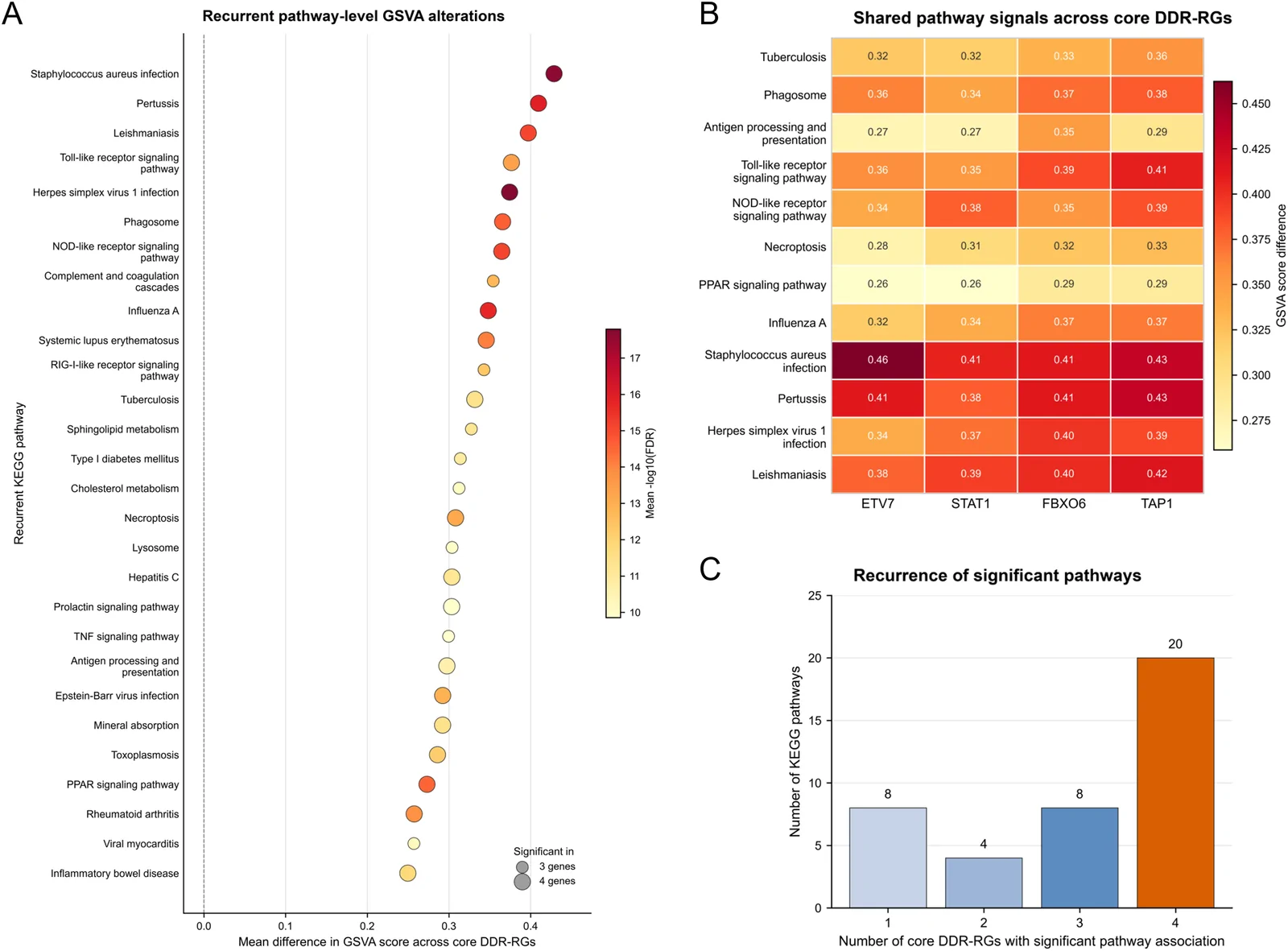
Gene set variation analysis (GSVA) of pathways associated with the core DNA damage repair-related genes (DDR-RGs). **(A)** Bubble plot summarizing recurrent pathway-level Kyoto Encyclopedia of Genes and Genomes (KEGG) pathway alterations across the four core DDR-RGs. Bubble size indicates whether a pathway was significant in three or four core genes, and color represents the mean -log10(FDR). **(B)** Heatmap showing shared pathway signals across ETV7, STAT1, FBXO6, and TAP1, with values indicating GSVA score differences. **(C)** Bar plot showing the recurrence of significant KEGG pathways according to the number of core DDR-RGs with significant pathway association.

### 3.7 Immune infiltration correlation analysis

Immune infiltration analysis using CIBERSORT revealed substantial remodeling of the immune microenvironment in TB patients (**Figure 8A**). Specifically, gamma-delta (γδ) T cells, monocytes, M1 macrophages, M2 macrophages, dendritic cells, eosinophils, and neutrophils were increased, whereas CD4^+^ T cells and CD8^+^ T cells were decreased (**Figure 8B**). Correlation analysis further showed that the expression levels of core DDR-RGs were significantly associated with multiple immune cell populations (**Figure 8C**), indicating associations between these genes and immune-cell infiltration patterns in TB.

**Figure 8:**
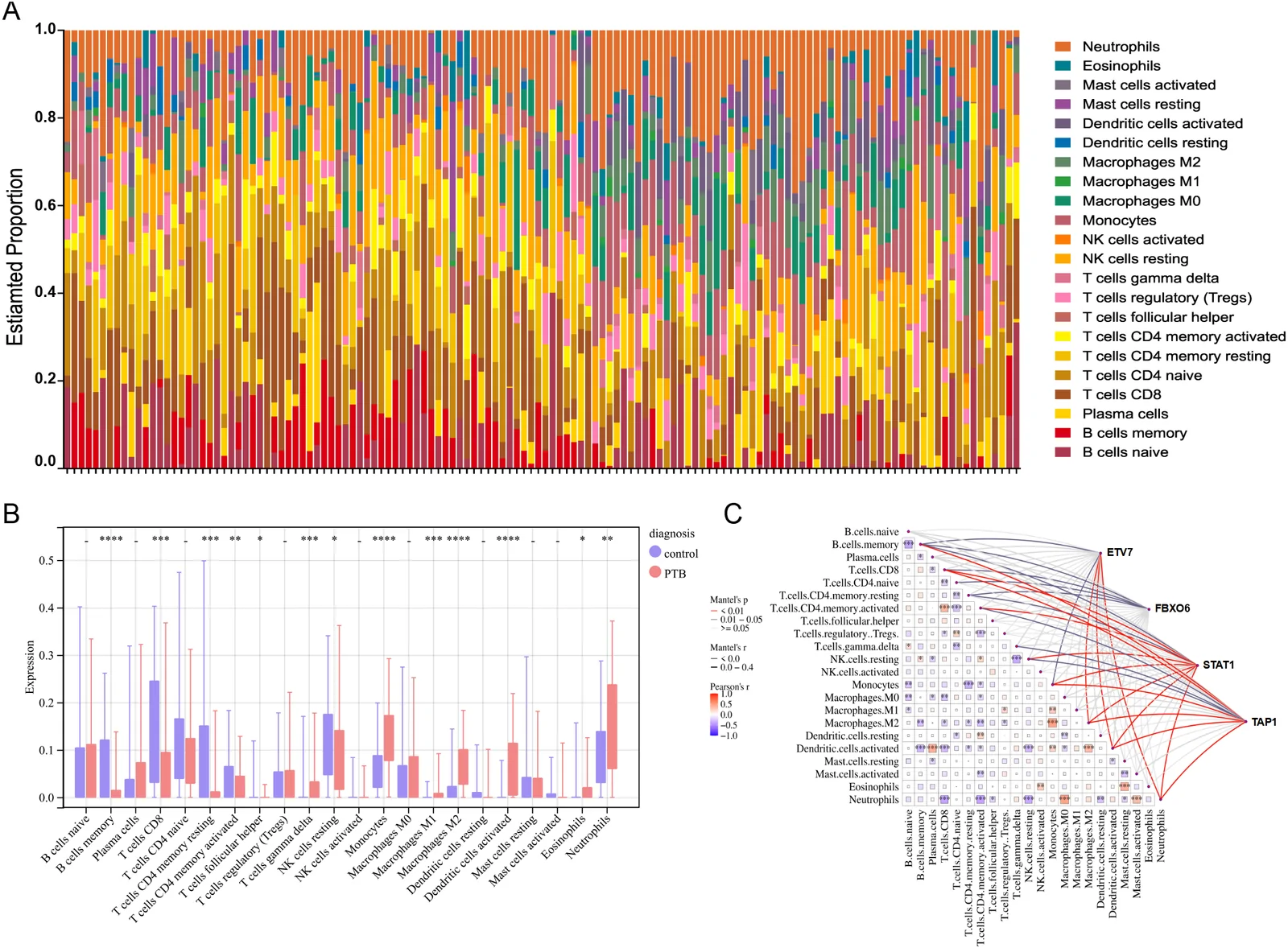
Immune infiltration analysis. **(A)** Heatmap showing the relative abundance of infiltrating immune cells in each sample. **(B)** Comparison of 22 immune cell types between tuberculosis (TB) and control samples. **(C)** Correlation network showing associations between the expression of core DNA damage repair-related genes (DDR-RGs) and immune cell infiltration levels. *P < 0.05, **P < 0.01, ***P < 0.001, and ****P < 0.0001.

### 3.8 Identification of DDR-related subtypes in TB

Consensus clustering based on the expression profiles of the four core DDR-RGs classified the 62 TB samples into two molecular subtypes. The optimal number of clusters was determined to be k = 2 according to the CDF curve, delta area plot, and consensus score (**Figures 9A–D**). UMAP showed clear separation between the two subtypes: Cluster 1, comprising 39 cases, and Cluster 2, comprising 23 cases (**Figure 9E**). The expression of all four core DDR-RGs was significantly higher in Cluster 1 than in Cluster 2 (**Figures 9F, G**). GSVA further showed that Cluster 1 was enriched in pathways including NOD-like receptor signaling, complement and coagulation cascades, necroptosis, RIG-I-like receptor signaling, TNF signaling, Toll-like receptor signaling, phagosome, and neutrophil extracellular trap formation (**Figure 9H**), indicating that Cluster 1 was characterized by stronger innate immune pathway activity. In addition, immune infiltration analysis showed that Cluster 1 had higher abundances of M2 macrophages, activated dendritic cells, and neutrophils but lower abundance of CD4^+^ T cells (**Figure 9I**).

**Figure 9:**
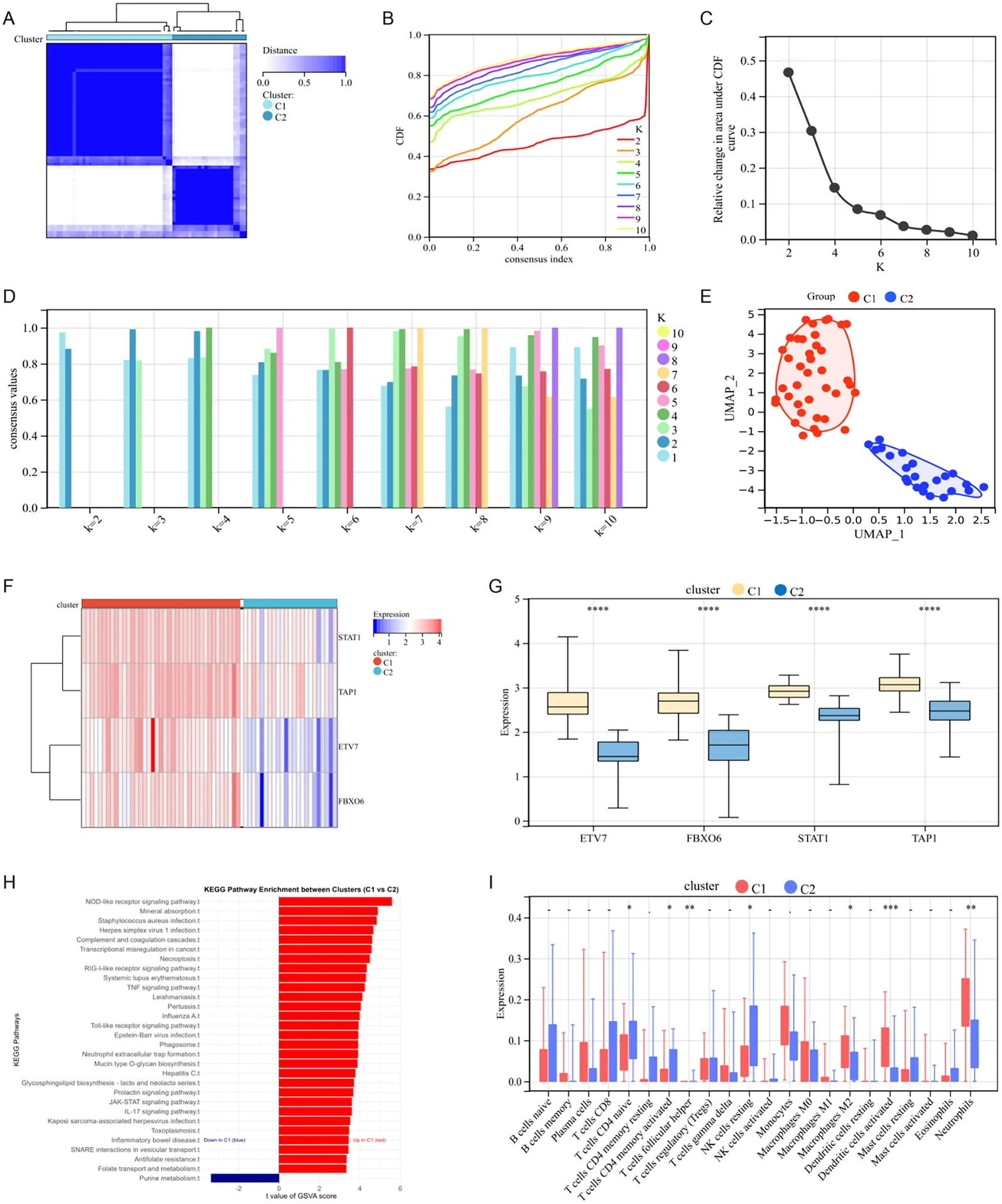
Molecular subtyping of tuberculosis (TB) based on the core DNA damage repair-related genes (DDR-RGs). **(A)** Consensus clustering matrix for k = 2. **(B)** Cumulative distribution function (CDF) curves for different values of k. **(C)** Delta area plot of the CDF curve. **(D)** Consensus scores for different clustering solutions. **(E)** Uniform manifold approximation and projection (UMAP) plot showing separation between Cluster 1 and Cluster 2. **(F)** Heatmap showing the expression profiles of the four core DDR-RGs in the two subtypes. **(G)** Box plots showing differential expression of the core DDR-RGs between the two subtypes. **(H)** Gene set variation analysis (GSVA)-based Kyoto Encyclopedia of Genes and Genomes (KEGG) pathway differences between the two subtypes. **(I)** Differences in immune cell infiltration between the two subtypes. *P < 0.05, **P < 0.01, ***P < 0.001.

### 3.9 Cell-type-specific expression patterns of core DDR-RGs at single-cell resolution in pulmonary TB

To elucidate the cellular localization of core DDR-RGs in pulmonary TB, integrated single-cell transcriptome analysis was performed using NC and ATB samples. UMAP-based clustering identified 12 distinct cell subpopulations, including CD4^+^ Naive/Mem T cells, CD4^+^ T cells (TCF7^+^), CD8^+^ Cytotoxic T cells, GammaDelta T cells, B Cells, Plasma lineage cells, CD14^+^ Monocytes, CD16^+^ Mono, cDC2, pDC, Neutrophils, and Platelets (**Figure 10A**). STAT1 and TAP1 exhibited broader expression across multiple immune cell subsets. In contrast, ETV7 and FBXO6 showed more recognizable cell-subset-biased spatial localization in the integrated UMAP space. ETV7 was mainly enriched in CD4^+^ Naive/Mem T cells, CD4^+^ T cells (TCF7^+^), CD8^+^ Cytotoxic T cells, and GammaDelta T cells (**Figure 10B**). FBXO6 was mainly enriched in T-cell subsets and monocyte-related subsets, especially CD14^+^ Monocytes and CD16^+^ Mono (**Figure 10C**). The split violin plots further showed the expression patterns of ETV7, FBXO6, TAP1, and STAT1 between NC and ATB groups across annotated cell subsets. ETV7 showed relatively low overall expression and was mainly detected in T-cell subsets, with slightly higher expression in ATB samples. FBXO6 exhibited higher expression than ETV7 and was more evident in T-cell subsets, monocyte subsets, dendritic-cell subsets, and Plasma lineage cells, particularly in the ATB group. TAP1 displayed a broader expression profile across T-cell subsets, monocyte subsets, cDC2, pDC, Plasma lineage cells, Neutrophils, and Platelets, suggesting its association with antigen-processing-related immune activation. STAT1 showed the broadest and most prominent expression pattern, with enhanced expression in ATB samples across T-cell subsets, monocyte subsets, cDC2, pDC, Neutrophils, B Cells, and Plasma lineage cells (**Figure 10D**). Collectively, these findings indicate that core DDR-RGs exhibit distinct cell-type-specific expression characteristics in pulmonary TB. ETV7 and FBXO6 show clearer subset-biased localization, mainly involving T-cell and monocyte-related immune compartments, whereas TAP1 and STAT1 display broader immune-cell distribution. These expression patterns were associated with T-cell responses, monocyte activation, antigen presentation, interferon signaling, and DDR-associated immune stress responses in active TB.

**Figure 10:**
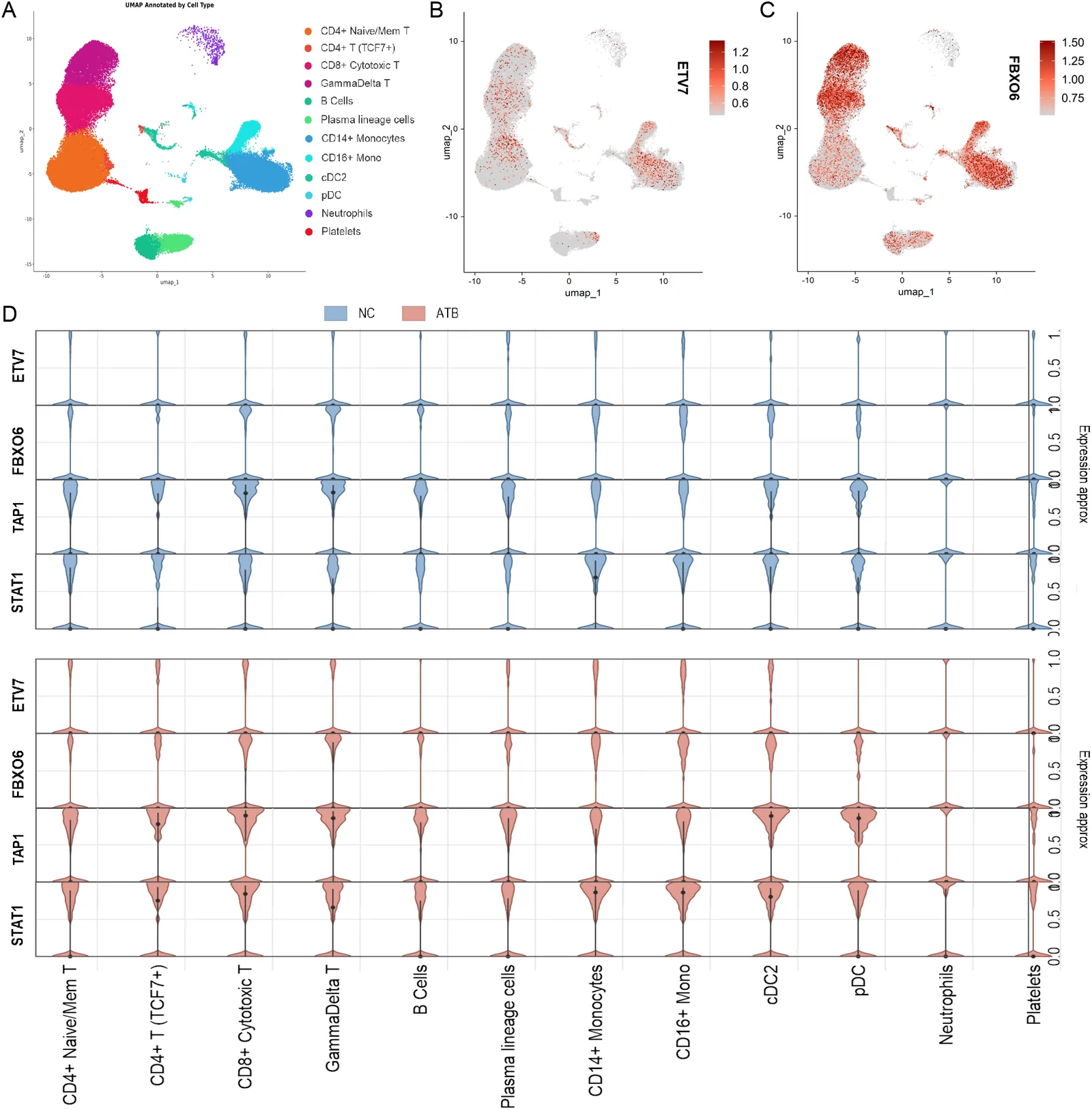
Distribution characteristics of core DNA damage repair-related genes (DDR-RGs) across cell subsets in the single-cell transcriptome of pulmonary tuberculosis. **(A)** UMAP visualization of integrated NC and ATB samples showing 12 annotated cell subpopulations. **(B-C)** Feature plots showing the spatial expression distribution of ETV7 **(B)** and FBXO6 **(C)**. **(D)** Split violin plots comparing the expression levels of ETV7, FBXO6, TAP1, and STAT1 between NC and ATB groups across the annotated cell subpopulations. Blue indicates NC samples, and red indicates ATB samples.

### 3.10 Candidate compounds from CTD screening

CTD-based screening identified 43 gene-compound interaction pairs involving the four core DDR-RGs and 35 candidate small-molecule compounds. Cytoscape was used to construct the gene-compound interaction network, which contained 39 nodes and 43 edges and summarized reported compound associations of the core DDR-RGs (**Figure 11**). Gene-level analysis showed that STAT1 had the highest number of associated compounds (26), including arsenic trioxide, ruxolitinib, and ivermectin. TAP1 and FBXO6 were each associated with eight compounds, whereas ETV7 was linked to only one candidate compound, estradiol. Further analysis identified six compounds reported to interact with two or more core DDR-RGs. Notably, estradiol interacted with ETV7, FBXO6, STAT1, and TAP1; arsenic trioxide and ivermectin were each associated with both STAT1 and TAP1; cyclosporine was associated with both FBXO6 and STAT1; and pirinixic acid and valproic acid were associated with both FBXO6 and TAP1. These results indicate that selected compounds warrant further evaluation in future host-directed studies.

**Figure 11:**
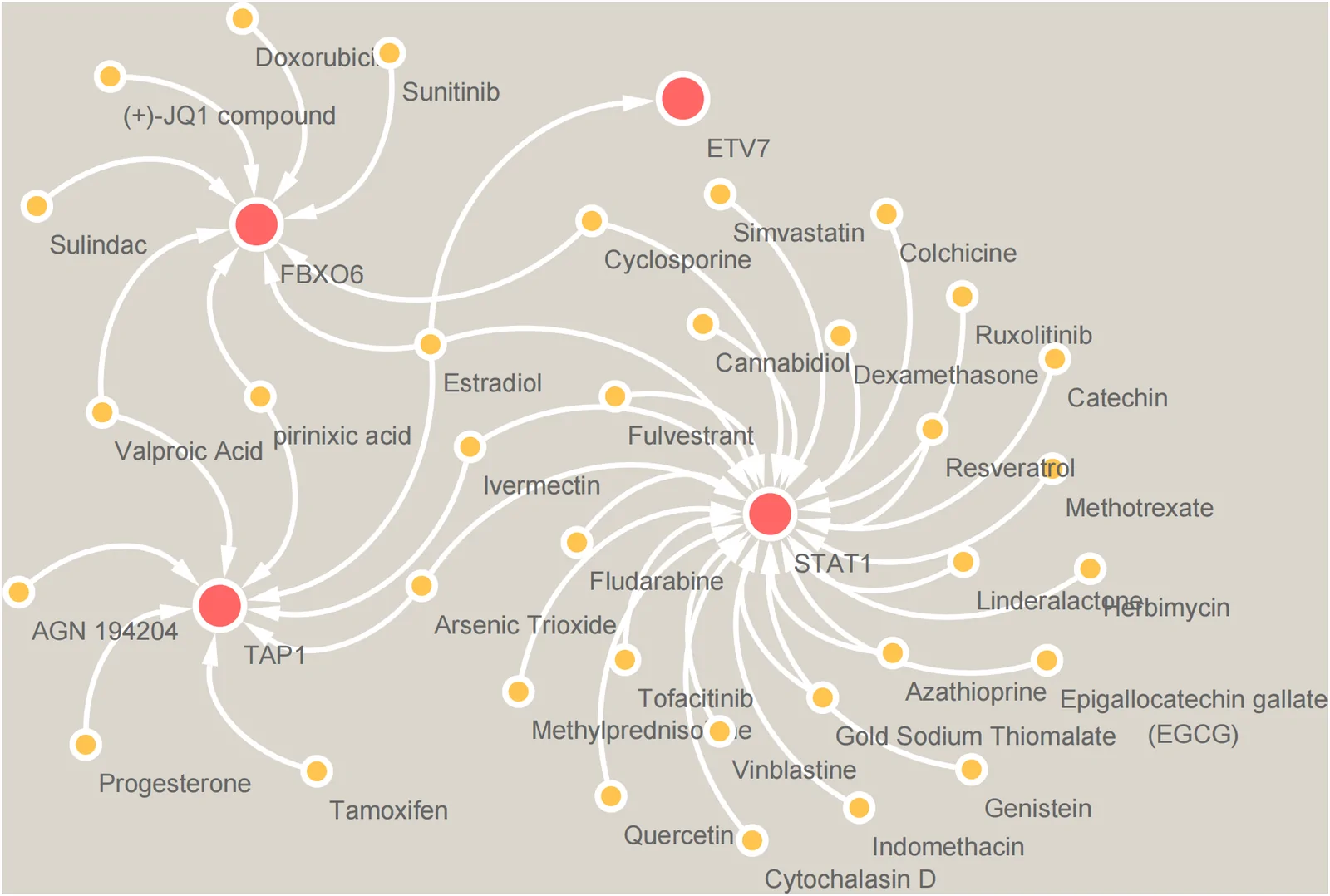
Gene-compound interaction network of the core DNA damage repair-related genes (DDR-RGs). Small-molecule compounds associated with the core DDR-RGs were identified from the Comparative Toxicogenomics Database (CTD), and the interaction network was visualized using Cytoscape. Nodes represent core DDR-RGs or candidate compounds, and edges represent potential interactions. The network includes four core DDR-RGs, 35 candidate compounds, and 43 gene-compound interaction pairs.

## 4 Discussion

TB remains one of the leading causes of death from infectious diseases worldwide (World Health Organization, 2025). Its characteristic latent infection state reflects a long-term and complex equilibrium between Mtb and host immunity (Cambier et al., 2014). Mtb employs multiple mechanisms to evade immune clearance, including inhibition of phagosome maturation, interference with antigen presentation, and modulation of inflammatory responses, and it can also influence host genomic homeostasis and stress-response networks (Cambier et al., 2014; Castro-Garza et al., 2018). DDR is a fundamental mechanism for maintaining genomic stability, mainly through pathways such as BER, NER, MMR, HR, and NHEJ (Sancar et al., 2004; Jackson and Bartek, 2009). In anti-TB immunity, DDR plays a dual role: appropriate repair supports the survival, proliferation, and effector function of immune cells, whereas dysregulated repair may lead to genomic instability, cellular senescence, or programmed cell death, thereby disrupting immune homeostasis (Sancar et al., 2004; Jackson and Bartek, 2009). In parallel, Mtb relies on an efficient DNA repair system to resist host oxidative stress and immune attack (Dos Vultos et al., 2009; Brzostek et al., 2014; Szulc-Kielbik et al., 2015; Reiche et al., 2017). Therefore, investigating TB from the perspective of DDR may inform disease diagnosis and host-response-focused research.

In this study, we integrated multiple GEO datasets and combined differential expression analysis, WGCNA, and machine learning strategies to identify four core DDR-RGs associated with pulmonary TB: ETV7, STAT1, FBXO6, and TAP1. Among the 110 model combinations evaluated, Ridge + SVM showed the best overall performance. The optimal model achieved AUC values of 0.991, 0.925, 0.939, and 0.981 across the training and validation cohorts, indicating robust discriminatory ability and possible clinical utility for pulmonary TB diagnosis. These findings are consistent with previous studies showing that active TB is characterized by interferon-inducible blood transcriptional signatures and that multicohort transcriptomic models can support pulmonary TB diagnosis (Berry et al., 2010; Sweeney et al., 2016). GSVA further showed that these core DDR-RGs were mainly enriched in immune-related pathways, including phagosome, PPAR signaling, NOD-like receptor signaling, Toll-like receptor signaling, necroptosis, and antigen processing and presentation (**Figure 7**). Immune infiltration analysis revealed increased proportions of monocytes, neutrophils, γδ T cells, and M1/M2 macrophages, together with decreased proportions of CD4^+^ T cells and CD8^+^ T cells in pulmonary TB (**Figure 8**). These observations suggest that DDR-related abnormalities are closely associated with immune microenvironment remodeling in TB. Consensus clustering based on the four core DDR-RGs further classified patients into two molecular subtypes (**Figure 9**). Cluster 1 showed higher expression of all core DDR-RGs and was characterized by stronger activation of innate immune pathways and increased infiltration of neutrophils, activated dendritic cells, and M2 macrophages, suggesting a molecular state with greater inflammatory activity and more substantial immune remodeling.

ETV7, a member of the E26 transformation-specific (ETS) transcription factor family, participates in interferon responses and inflammatory regulation (Froggatt et al., 2021). Although ETV7 is not a classical DNA repair enzyme, its reported involvement in interferon-related antiviral transcriptional regulation (Froggatt et al., 2021) and its association with DDR-related immune programs in the present study support a potential link between ETV7, inflammatory signaling, and DNA damage-associated stress responses in TB. In this study, ETV7 was significantly upregulated in pulmonary TB, suggesting a role in host anti-infection immunity through integration of interferon signaling and DNA damage-associated stress responses. scRNA-seq analysis showed that ETV7 expression was relatively restricted and was primarily localized to CD4^+^ naive/memory T cells, TCF7^+^ CD4^+^ T cells, CD8^+^ cytotoxic T cells, and γδ T cells. Scattered expression was also observed in CD14^+^ monocytes, CD16^+^ monocytes, and a small number of plasma lineage cells, with a slight increase in positive cells in the ATB group. This distribution suggests that ETV7 may be more closely involved in T-cell-related transcriptional regulation and stress responses, while also participating in selected monocyte- and plasma-lineage-associated immune processes.

STAT1 is the central transcription factor of the JAK-STAT pathway and a critical downstream effector of interferon-gamma-mediated antimycobacterial immunity (Flynn et al., 1993; Stark and Darnell, 2012). In TB, STAT1 activation is closely linked to interferon-dependent antimicrobial responses and inflammatory mediator expression (Flynn et al., 1993; Berry et al., 2010; Stark and Darnell, 2012). STAT1 also exhibits cross-talk with DNA damage-associated stress signaling, as STAT1 can interact with p53 to enhance DNA damage-induced apoptosis (Townsend et al., 2004). In this study, STAT1 was significantly upregulated and showed the broadest distribution at single-cell resolution. It was mainly expressed in CD4^+^ naive/memory T cells, TCF7^+^ CD4^+^ T cells, CD8^+^ cytotoxic T cells, γδ T cells, CD14^+^ monocytes, CD16^+^ monocytes, cDC2, pDC, neutrophils, B cells, and plasma lineage cells, with overall enhancement in the ATB group. These findings indicate that STAT1 was broadly associated with T-cell responses, monocyte activation, dendritic-cell-associated antigen presentation, neutrophil inflammation, and DNA damage-associated stress in pulmonary TB.

FBXO6 functions as a substrate-recognition component of the SKP1-CUL1-F-box (SCF)-type E3 ubiquitin ligase complex and is mainly involved in ubiquitin-mediated protein degradation (Glenn et al., 2008). Ubiquitination is an essential regulatory process influencing checkpoint proteins, repair proteins, and inflammatory signaling molecules within the DDR network (Glenn et al., 2008; Jackson and Bartek, 2009; Zhang et al., 2009). Previous studies have suggested that FBXO6 can affect DNA damage-associated signaling and cell fate by modulating Chk1 stability and cellular sensitivity to replication stress (Zhang et al., 2009). In the present study, FBXO6 was significantly upregulated in pulmonary TB, consistent with a possible role in ubiquitination-related regulation of DDR-associated proteins and immune signaling molecules. scRNA-seq analysis showed that FBXO6 was mainly distributed in CD4^+^ naive/memory T cells, TCF7^+^ CD4^+^ T cells, CD8^+^ cytotoxic T cells, γδ T cells, CD14^+^ monocytes, CD16^+^ monocytes, and plasma lineage cells, with fewer positive signals in cDC2, pDC, neutrophils, and platelets. This pattern links FBXO6 expression to T-cell-related stress and ubiquitination networks, as well as monocyte-, dendritic-cell-, plasma-lineage-, and platelet-associated immune features in TB.

TAP1 is a key component of the transporter associated with antigen processing complex and is responsible for delivering cytoplasmic antigen peptides into the endoplasmic reticulum for loading onto MHC class I molecules, thereby promoting CD8^+^ T-cell recognition and cytotoxic responses (Eggensperger and Tampé, 2015). Beyond its established role in antigen processing (Eggensperger and Tampé, 2015), TAP1 was associated in the present study with immune- and DDR-related pathway activity, supporting a contextual connection between antigen presentation and DNA damage-associated immune stress in TB. In this study, TAP1 was significantly upregulated in pulmonary TB, suggesting enhanced antigen processing and presentation during infection. Single-cell analysis further showed that TAP1 was widely expressed in CD4^+^ naive/memory T cells, TCF7^+^ CD4^+^ T cells, CD8^+^ cytotoxic T cells, γδ T cells, CD14^+^ monocytes, CD16^+^ monocytes, and plasma lineage cells, with scattered expression in cDC2, pDC, neutrophils, and platelets. These findings indicate that TAP1 is linked not only to adaptive immune antigen presentation but also to persistent antigenic stimulation and inflammatory activation across T-cell, monocyte, dendritic-cell, neutrophil, and plasma-lineage compartments in pulmonary TB.

Single-cell transcriptome analysis further supported the expression patterns of core DDR-RGs at cellular resolution (**Figure 10**). Integrated analysis of NC and ATB samples identified 12 distinct cell subpopulations, including CD4^+^ naive/memory T cells, TCF7^+^ CD4^+^ T cells, CD8^+^ cytotoxic T cells, γδ T cells, B cells, plasma lineage cells, CD14^+^ monocytes, CD16^+^ monocytes, cDC2, pDC, neutrophils, and platelets. Dot and violin plots showed that ETV7, FBXO6, STAT1, and TAP1 exhibited overall higher expression in the ATB group, with the ATB1 sample showing the most pronounced increase. Importantly, these genes showed distinct cell-subset-specific expression patterns: ETV7 was relatively restricted to T-cell subsets, with scattered expression in CD14^+^ monocytes, CD16^+^ monocytes, and plasma lineage cells; FBXO6 was mainly expressed in T-cell subsets, monocyte subsets, and plasma lineage cells, with fewer signals in cDC2, pDC, neutrophils, and platelets; STAT1 showed the broadest distribution across T-cell subsets, monocytes, dendritic-cell subsets, neutrophils, B cells, and plasma lineage cells; and TAP1 was broadly expressed in T-cell subsets, monocytes, plasma lineage cells, and scattered cDC2, pDC, neutrophils, and platelets. These results suggest that DDR-related abnormalities in pulmonary TB are not merely reflected by increased bulk transcript levels but are concentrated within key effector-cell populations in the immune microenvironment.

GSVA suggested that core DDR-RGs were mainly associated with pathways such as phagosome, PPAR signaling, NOD-like receptor signaling, Toll-like receptor signaling, necroptosis, and antigen processing and presentation, all of which are closely linked to host stress responses and inflammation (**Figure 7**) (Jackson and Bartek, 2009; Cambier et al., 2014). For example, excessive reactive oxygen species generated during pathogen recognition and inflammatory amplification can induce oxidative DNA damage and activate repair pathways (Sancar et al., 2004; Jackson and Bartek, 2009). Persistent DNA damage stress may in turn contribute to tissue injury through cell death programs and inflammatory cascades (Sancar et al., 2004; Jackson and Bartek, 2009). Immune infiltration analysis further showed that ETV7, STAT1, FBXO6, and TAP1 were positively correlated with monocytes, macrophages, and neutrophils and negatively correlated with CD8^+^ T cells. Together with the single-cell results, these findings indicate associations between DDR-related molecules and immune microenvironmental features involving both T cells and myeloid cells.

The molecular subtype analysis further highlighted substantial DDR-related heterogeneity in pulmonary TB (**Figure 9**). Cluster 1 showed higher overall expression of the four core DDR-RGs and was significantly enriched in inflammatory pathways, including NOD-like receptor signaling, complement and coagulation cascades, necroptosis, RIG-I-like receptor signaling, TNF signaling, Toll-like receptor signaling, phagosome, and neutrophil extracellular trap formation. This pattern was accompanied by increased infiltration of M2 macrophages, activated dendritic cells, and neutrophils. These findings indicate that Cluster 1 was characterized by stronger immune activation, greater inflammatory burden, and more pronounced DDR-related stress. In contrast, Cluster 2 appeared to be associated with a relatively low-inflammation and weak-stress state. This classification may provide a framework for future risk-stratification studies and host-response-guided research in pulmonary TB.

In addition to their diagnostic and subtyping value, the core DDR-RGs showed multiple reported compound associations based on CTD screening (**Figure 11**) (Davis et al., 2025). We identified 43 gene-compound interaction pairs involving 35 candidate small-molecule compounds. The resulting interaction network indicated that these genes have multiple reported compound associations in CTD. STAT1 was associated with the greatest number of candidate compounds, followed by TAP1 and FBXO6, whereas ETV7 was linked to only one compound. Six compounds were reported to interact with two or more core DDR-RGs. For example, estradiol interacted with ETV7, FBXO6, STAT1, and TAP1; arsenic trioxide and ivermectin were each associated with both STAT1 and TAP1; and cyclosporine, pirinixic acid, and valproic acid also showed associations with multiple core genes. These findings indicate shared compound-association patterns among the core DDR-RGs and support further evaluation of selected compounds in future host-directed studies (Wallis and Hafner, 2015).

This study has several limitations. First, the DDR-RG set was derived mainly from database-based screening and may therefore be affected by selection bias. Second, the public datasets were generated from specific populations and platforms, which may limit generalizability across regions, ethnicities, and disease stages. Third, although scRNA-seq analysis provided cell-type-specific expression patterns, functional evidence showing how core DDR-RGs regulate DNA damage-associated stress and immune responses in specific cell populations remains lacking. Fourth, cell-cell communication patterns were not assessed in this study, which limited direct evaluation of ligand-receptor interactions among immune subsets. Future work integrating CellChat-based communication analysis, spatial transcriptomics, and functional validation may help define how DDR-related signals are coordinated across immune compartments in TB. Fifth, the candidate small-molecule compounds identified through CTD screening are preliminary and require pharmacologic and translational validation in cellular models, animal studies, and clinical cohorts (Davis et al., 2025). Future studies combining multicenter prospective cohorts, longitudinal sampling, interpretable machine learning/deep learning models, and functional experiments will be important for evaluating the diagnostic robustness and biological relevance of ETV7, STAT1, FBXO6, and TAP1 in pulmonary TB.

In summary, this study systematically identified four core DDR-RGs associated with pulmonary TB, namely ETV7, STAT1, FBXO6, and TAP1. A machine learning model with strong diagnostic performance was established, and multidimensional analyses further showed that these genes are predominantly expressed in key immune-cell subsets, including CD4^+^ naive/memory T cells, TCF7^+^ CD4^+^ T cells, CD8^+^ cytotoxic T cells, γδ T cells, B cells, plasma lineage cells, CD14^+^ monocytes, CD16^+^ monocytes, cDC2, pDC, neutrophils, and platelets. These findings suggest that the identified genes are associated with T-cell responses, monocyte activation, dendritic-cell-associated antigen presentation, interferon responses, and DDR-related immune regulation in pulmonary TB. In addition, candidate small-molecule compounds identified through CTD analysis provide leads for future host-directed research. Overall, this study advances understanding of DDR-related molecular programs in pulmonary TB from the perspectives of diagnostic modeling, cell-type-specific expression patterns at single-cell resolution, molecular subtyping, immune heterogeneity, and compound-association screening, supporting future work on diagnosis, molecular stratification, and host-response-guided research.

## Data Availability

The public datasets analyzed in this study are available from the Gene Expression Omnibus database under accession numbers GSE83456, GSE152532, GSE19444, GSE19491, and GSE54992. The single-cell RNA-seq datasets generated in this study have been deposited in the China National GeneBank Nucleotide Sequence Archive under BioProject accession number PRJCA067981 https://ngdc.cncb.ac.cn/gsub/submit/bioproject/PRJCA067981. The analysis scripts used to reproduce the main computational analyses are available in Zenodo at https://doi.org/10.5281/zenodo.21335723. The original contributions presented in this study are included in the article and **Supplementary Materials**.

## Supplementary material

Supplementary Figure 1 Principal component analysis (PCA) plots showing sample clustering before and after ComBat batch correction.

Supplementary Figure 2 Gene dendrogram and module assignment identified by hierarchical clustering in weighted gene co-expression network analysis (WGCNA). The upper panel shows the gene clustering dendrogram, and the color bars indicate dynamic tree cut and merged module assignments.

Supplementary Table 1 GEO datasets used in this study.

Supplementary Table 2 DNA damage repair-related genes retrieved from the GeneCards database.

Supplementary Table 3 Differentially expressed genes between tuberculosis patients and healthy controls.

Supplementary Table 4 Genes included in the key WGCNA module.
